# PFKFB3 Inhibitor 3PO Reduces Cardiac Remodeling after Myocardial Infarction by Regulating the TGF-β1/SMAD2/3 Pathway

**DOI:** 10.3390/biom13071072

**Published:** 2023-07-03

**Authors:** Qian Yang, Xiao Zong, Lingfang Zhuang, Roubai Pan, Xierenayi Tudi, Qin Fan, Rong Tao

**Affiliations:** 1 Department of Cardiovascular Medicine, Ruijin Hospital, Shanghai Jiao Tong University School of Medicine, Shanghai 200025, China; 2 Institution of Cardiovascular Diseases, Shanghai Jiao Tong University School of Medicine, Shanghai 200025, China

**Keywords:** PFKFB3, myocardial infarction (MI), cardiac remodeling, cardiac fibroblast, fibrosis

## Abstract

Adverse cardiac remodeling, including cardiac fibrosis, after myocardial infarction (MI) is a major cause of long-term heart failure. 6-phosphofructo-2-kinase/fructose-2,6-biphosphatase 3 (PFKFB3), an enzyme that regulates glucose metabolism, also plays an important role in various fibrotic and cardiovascular diseases. However, its effects on MI remain unknown. Here, PFKFB3 inhibitor 3-(3-pyridinyl)-1-(4-pyridinyl)-2-propen-1-one (3PO) and a permanent left anterior descending ligation mouse model were used to explore the functional role of PFKFB3 in MI. We showed that PFKFB3 expression increased significantly in the area of cardiac infarction during the early phase after MI, peaking on day 3. 3PO treatment markedly improved cardiac function, accompanied by decreased infarction size and collagen density in the infarct area. Meanwhile, 3PO attenuated cardiac fibrosis after MI by reducing the expression of collagen and fibronectin in murine hearts. Notably, 3PO reduced PFKFB3 expression and inhibited the transforming growth factor-beta 1/mothers against the decapentaplegic homolog 2/3 (TGF-β1/SMAD2/3) signaling pathway to inhibit cardiac fibrosis after MI. Moreover, PFKFB3 expression in neonatal rat cardiac fibroblasts (NRCFs) increased significantly after MI and under hypoxia, whereas 3PO alleviated the migratory capacity and activation of NRCFs induced by TGF-β1. In conclusion, 3PO effectively reduced fibrosis and improved adverse cardiac remodeling after MI, suggesting PFKFB3 inhibition as a novel therapeutic strategy to reduce the incidence of chronic heart failure following MI.

## 1. Introduction

As a leading global cause of damage to human health, cardiovascular disease places a heavy economic burden on society and impairs overall quality of life [1]. In particular, cardiac fibrosis after myocardial infarction (MI), a prevalent form of cardiovascular illness [2], causes detrimental cardiac remodeling in the later stages, triggering ventricular systolic and diastolic dysfunction and ultimately ending in heart failure [3]. It is therefore crucial to implement therapies that effectively prevent harmful cardiac remodeling processes in patients with MI.

Resident cardiac fibroblasts (CFs) account for approximately 20% of the non-myocytes in the normal murine heart [4]. The inflammatory, proliferative, and maturation phases are the three stages of the healing process following MI [5]. From the proliferative stage after MI, when cardiomyocytes rapidly and dramatically necrose, CFs in the infarct and surrounding areas begin to multiply in large numbers and undergo a transformation into secretory and contractile cells, known as myofibroblasts, which express alpha-smooth muscle actin (α-SMA) [5,6,7,8]. Reparative fibrosis, which is crucial for the recovery of infarcted myocardium and structural integrity of the ventricular wall in the early stages of infarction, is the process by which myofibroblasts produce large amounts of collagen, fibronectin, and non-structural extracellular matrix to replace necrotic tissue and form fibrotic scars [9]. However, severe fibrotic remodeling causes systolic and diastolic dysfunction by increasing wall stiffness and decreasing ventricular compliance.

The transforming growth factor-beta 1 (TGF-β1), a pro-fibrotic growth factor, was shown to be highly expressed in the infarct area of the heart on day 3 after MI, which is during the proliferative phase [10]. Following increased production in the infarcted and border regions, TGF-β1 binds to TGF-β receptor on the cell membrane and phosphorylates mothers against the decapentaplegic homolog 2 (SMAD2) and SMAD3, allowing these to bind SMAD4 to form a complex in which SMAD7 functions as an inhibitory molecule [11]. The TGF-β1/SMAD2/3 signaling pathway is critical for the development of cardiac fibrosis after MI [12]. TGF-β1 can regulate the transcription of protein target genes of the pro-fibrotic response and promote the activation of cardiac fibroblast and protein synthesis of the cardiac extracellular matrix by activating the TGF-β1/SMAD2/3 signaling pathway, which affects the process of cardiac fibrosis and cardiac remodeling in the late stages of MI [5,13]. Inhibition of the TGF-β1/SMAD2/3 signaling pathway has been shown in numerous studies to be effective in preventing cardiac fibrosis following MI [14,15,16,17].

6-phosphofructo-2-kinase/fructose-2,6-biphosphatase 3 (PFKFB3) is an intracellular protein that is frequently overexpressed in numerous human tumors but is expressed at low levels in normal tissue [18]. PFKFB3, a member of the PFKFB isoenzyme family, is a bifunctional enzyme that regulates glycolysis by controlling cytoplasmic levels of fructose-2,6-bisphosphate (Fru-2,6-BP) [18,19]. PFKFB3 also plays important roles in promoting cell cycle progression, regulating autophagy, and inhibiting apoptosis [20,21]. Hypoxia induces increased expression of PFKFB3, which promotes cell proliferation and migration [22,23]. Previous studies have shown that PFKFB3 has detrimental effects on a variety of cardiovascular diseases, such as pulmonary hypertension and atherosclerosis [24,25,26]. Alternatively, 3-(3-pyridinyl)-1-(4-pyridinyl)-2-propen-1-one (3PO), a specific inhibitor of PFKFB3, has been involved in many types of research related to PFKFB3. For tumor research, 3PO can decrease intracellular Fru-2,6-BP and suppress glycolytic flux in transformed cells [27]. 3PO can reduce the invasion, intravasation, and metastasis of cancer cells [28]. In addition, 3PO also has significant effects on several fibrotic diseases such as pulmonary fibrosis and liver fibrosis. Hu et al. [29] demonstrated that 3PO inhibited collagen synthesis in lung fibroblasts. Mejias et al. [30] found that 3PO markedly prevented the activation of hepatic stellate cells and the extent of fibrosis after liver damage. Nevertheless, it remains unknown whether 3PO can reduce excessive cardiac fibrosis and improve adverse ventricular remodeling after MI.

In the present study, we elucidated the contribution of PFKFB3 to MI by detecting the expression of PFKFB3 in murine ischemic myocardium. We found that treatment with 3PO reduced fibrotic remodeling after MI by regulating the TGF-β1/SMAD2/3 pathway. Furthermore, we demonstrated that PFKFB3 is expressed largely in CFs and that 3PO attenuated the migration, proliferation, and activation of CFs induced by TGF-β1.

## 2. Materials and Methods

### 2.1. Mice

All animal experiments were conducted in accordance with the approval of the Experimental Animal Ethical Committee of Ruijin hospital. Male mice were used in this study based on previous studies showing that estrogen protects against the development of ischemic cardiomyopathy [31,32].

Wild-type (WT) C57BL/6 mice (male, 8–10 weeks old; SLRC Laboratory Animals, Shanghai, China) were used. Mice were randomly divided into four treatment groups: dimethyl sulfoxide (DMSO), 3PO, DMSO + MI, and 3PO + MI. To effect PFKFB3 inhibition, the small molecule inhibitor 3PO (875 μg/day, dissolved in 250 μL DMSO; HY-19824, MCE) was administered by intraperitoneal (i.p.) injection every other day following MI surgery. Based on the usage of 3PO in other animal disease models, reagent instructions and the outcome of pre-experiments, the dose and treatment times have been determined [30,33,34]. Control mice received corresponding DMSO i.p. injections following the sham operation. Mice were sacrificed by spinal cord dislocation, followed by heart harvest at −80 °C conditions for subsequent analyses.

### 2.2. Isolation and Culture of Neonatal Rat Cardiac Fibroblasts (NRCFs)

All animal experiments were conducted in accordance with the approval of the Experimental Animal Ethical Committee of Ruijin hospital. Isolation of NRCFs from the hearts of neonatal Sprague Dawley rats (1–3 days postnatal) was performed as previously described [35]. After disinfection, the hearts of neonatal rats were removed and washed twice, minced into 4 mm^3^ pieces, and digested using 0.04% trypsin buffer at 4 °C overnight. The tissue was digested 6 times with Hank’s balanced salt solution containing 0.1% (1 mg/mL) collagenase II (#LS004176; Worthington Biochemical, Lakewood, NJ, USA) in a 37 °C water bath after aspirating the supernatant. Each time, the digestate was collected and replaced.

During each digestion cycle, the supernatant was collected and digestion was stopped by adding 7 mL 10% Dulbecco’s modified Eagle medium (DMEM) high-glucose medium. After the end of the digestion process, the supernatant was passed through 70 μm cell strainers, centrifuged at 1000 rpm for 5 min to collect cell pellets, and resuspended in DMEM high-glucose medium containing 10% fetal bovine serum, 100 U/mL penicillin, and 100 U/mL streptomycin. The cells were then seeded in 10 cm^2^ culture dishes and placed in a culture incubator with 95% air and 5% CO_2_ at 37 °C for 1.5 h. After differential adhesion, the supernatant was removed and the fibroblasts at the bottom of the culture dishes were cultured in 10% DMEM high-glucose medium for further study.

### 2.3. Induction of MI

Mice in the experimental treatment groups underwent permanent left anterior descending (LAD) artery ligation or sham operation without ligation, as previously described [36]. In brief, mice were lightly anesthetized with 2% isoflurane, intubated with a 22-gauge tube, and mechanically ventilated using a small rodent respirator. After placing the mouse in a right lateral decubitus position, a 0.5–1 cm skin incision was made on the left chest, the muscle layer was separated, and the chest was opened between three or four ribs. The LAD segment that corresponds to roughly 1.5 to 2 mm lower than the tip of the left auricle was permanently ligated with an 8–0 monofilament nylon suture once the left ventricle and left auricle were made visible. The chest wall and skin incisions were then stitched together using a 5–0 suture. Mice that died within the first 24 h following surgery were not included in the analysis. The sham-operated group underwent comparable surgeries without arterial ligation.

### 2.4. Western Blotting

Heart tissue samples or cultured cells were homogenized in RIPA solution (50 mM Tris·HCl, 150 mM NaCl, 1% Triton X-100, 1% sodium deoxycholate, 0.1% sodium dodecyl sulfate, and sodium orthovanadate) supplemented with phenylmethanesulfonyl fluoride and Complete Mini protease inhibitor cocktail (Catalog #11836153001, Roche, Madison, WI, USA). In vivo, tissue samples were homogenized using a homogenizer. Protein concentration was determined using a bicinchoninic acid protein assay kit (Catalog #CW-0014S, CWBio, Beijing, China). Total proteins extracted from heart samples or cells were separated on 7.5, 10, or 12.5% sodium dodecyl sulfate-polyacrylamide electrophoresis gels (EpiZyme, Cambridge, MA, USA) and transferred to polyvinylidene fluoride membranes (Millipore, Billerica, MA, USA). The membranes were blocked with 5% non-fat milk or 5% bovine serum albumin (for phosphorylated proteins) in Tris-buffered saline with 0.1% Tween 20 for 1.5 h at room temperature, incubated overnight with primary antibodies at 4 °C, and incubated with horseradish peroxidase-conjugated secondary antibody (1:3000) for 70 min at room temperature. Subsequently, specific bands were detected using an enhanced chemiluminescence reagent (Catalog #180-5001, Tanon, Shanghai, China), and the obtained images were analyzed using ImageJ software (National Institutes of Health, Bethesda, MD, USA). The primary antibodies used in this study are provided in Table S1.

### 2.5. Immunofluorescence Analyses

Immunohistochemistry of paraffin-embedded sections of the heart tissue samples fixed with 4% paraformaldehyde (PFA) was performed as previously described [37]. Briefly, after deparaffinization, rehydration, and heat-mediated antigen retrieval (Catalog #G1202-250 ML, Servicebio, Wuhan, China), sections were blocked with 1x phosphate-buffered saline (PBS) containing 5% bovine serum albumin and then incubated with the appropriate primary antibodies at 4 °C overnight. The primary antibodies used are provided in Table S1. Negative controls using IgG isotype control antibodies were used to validate primary antibody specificity. Particularly in double-staining experiments, suitable Alexa Fluor-coupled secondary antibodies were used, being incubated for 1 h at room temperature. The sections were counterstained with 4′,6-diamidino-2-phenylindole (DAPI) (Catalog #C1006, Beyotime Biotechnology, Shanghai, China) and then coverslipped, followed by observation with an Olympus fluorescence microscope (Tokyo, Japan). ImageJ software was used to analyze the images. A representative image for each group was chosen based on the mean values.

### 2.6. Immunocytochemistry

In 6-well culture plates, the right number of NRCFs was seeded. After stimulation, the supernatant was removed and the cells were washed three times with phosphate-buffered saline for 5 min. Subsequently, the cells were fixed with PFA at 37 °C for 30 min, washed again, and permeabilized with 0.1% Triton X-100 (Beyotime Biotechnology, Shanghai, China) for 15 min at room temperature. The following steps were similar to those for the immunofluorescence of paraffin-embedded slices, as previously described. The primary antibodies used are provided in Table S1.

### 2.7. Pathological Staining and TdT-Mediated dUTP Nick-End Labeling (TUNEL) Staining

Mouse heart samples were fixed using 4% PFA, embedded in paraffin, and cut into 5–6 µm transverse sections at the papillary muscle level. To measure the degree of cardiac fibrosis, paraffin-embedded sections at the papillary level were stained with Masson’s trichrome and picrosirius red stains and then evaluated under regular polychromatic illumination. ImageJ software was used to analyze the images. The ratio of the scar area to the overall section area was used to measure infarction size. The ratio of the positively stained area to the overall scar area was used to calculate collagen density. For apoptosis analysis of cardiomyocytes, paraffin-embedded sections were co-stained with TUNEL stains (Beyotime Biotechnology, Shanghai, China) and α-actinin antibody (Catalog #GB11555, Servicebio, Wuhan, China) following the manufacturer’s protocol. The images were captured using a fluorescence microscope and analyzed by ImageJ software. The TUNEL-positive rate was calculated and represented as the number of TUNEL-positive cells (among the α-actinin-positive cells) divided by the total number of nuclei.

### 2.8. Echocardiography

Transthoracic echocardiography was performed using a Vevo 2100 instrument (VisualSonics, Toronto, ON, Canada) equipped with an MS-400 imaging transducer. Mice were briefly anesthetized with isoflurane before being placed in the supine position on an echo pad. To reduce data variation, mice were anesthetized throughout the echocardiographic examination, and the heart rate of each mouse was maintained between 450 and 500 bpm. To directly assess the left ventricular (LV) end-diastolic volume and LV end-systolic volume, B-mode tracings of the LV endocardial boundary in a parasternal long axis were performed. M-mode tracings were recorded through the anterior and posterior LV walls at the papillary muscle level in the same long-axis view to measure the interventricular septum (IVS) and posterior wall (PW) dimensions. An echocardiographer blinded to the therapy groups acquired and analyzed the echocardiograms.

### 2.9. Real-Time Quantitative Polymerase Chain Reaction (RT-qPCR)

Total RNA samples from isolated murine heart tissues and cultured cells were prepared using TRIZOL reagent (Invitrogen, Thermo Fisher, Carlsbad, CA, USA), according to the manufacturer’s instructions. The first-strand cDNA synthesis kit (ABclonal, Cambridge, MA, USA) was used for cDNA synthesis. The resulting cDNA fragments were amplified in an RT-qPCR machine (Applied Biosystems Q6, Foster City, CA, USA) using an SYBR Green PCR kit (Catalog #Q711-02/03, Vazyme, Jiangsu, China). Real-time cycler conditions were as follows: initial activation step (95 °C for 30 s); 2-step cycling (denaturation 95 °C for 10 s; combined annealing/extension 60 °C for 30 s) × 40 cycles. The relative mRNA expression or fold change of each target gene was computed using the 2^–∆∆Ct^ method after the mRNA level was normalized to that of endogenous *Gapdh* expression. Primer sequences for the genes of interest are provided in Table S3.

### 2.10. Migration and Proliferation Evaluation of NRCFs with Stimulation

NRCFs were seeded in 6-well culture plates for hypoxia and TGF-β stimulation experiments. Before stimulation, the cells were starved in DMEM high-glucose medium containing 1% fetal bovine serum, 100 U/mL penicillin, and 100 U/mL streptomycin for 24 h. For the hypoxia stimulation experiment, cells were treated under hypoxia for 0, 6, 12, and 24 h. For TGF-β stimulation experiments, cells were treated with or without 20 ng/mL TGF-β (R&D Systems, Minneapolis, MN, USA) for 24 h in the absence or presence of 3PO (30 μM for 1 h prior to TGF-β or vehicle administration). Cells were then harvested for further analysis.

The migration capacity of NRCFs was evaluated using a scratch assay and a Boyden chamber assay. As for the scratch assay, a wound area was created by scraping the bottom of each well with a 200 μL pipette tip. The percentage of the initial scratch area at 0 h that was repopulated by fibroblasts after 24 h was used to calculate the migration capacity of the fibroblasts. With regard to the Boyden chamber assay, NRCFs were seeded onto 8 μm pore culture inserts (Millipore, Billerica, MA, USA) in a 24-well culture plate with cell culture medium containing a stimulation substance in the lower chamber. The medium was taken out of the wells after 48 h, and the cells were washed with PBS and fixed with 4% PFA for 30 min. Following that, each well was provided 0.2% crystal violet for 20 min to stain the cells that had crossed the membrane. The cells were stained, washed with PBS, and then photographed under an optical microscope. The positively stained cells were subsequently counted using ImageJ software.

The proliferation capacity was evaluated using a 5-Ethynyl-2’-deoxyuridine (EDU) Cell Proliferation Detection Kit (Ribo Biotechnology, Guangzhou, China). NRCFs were seeded in 24-well culture plates and a TGF-β stimulation experiment was conducted for 24 h. According to the manufacturer’s instructions, each well was added with EDU-containing medium for 2 h, after which the cells were fixed and stained with relevant reagents. The images were captured using a fluorescence microscope and analyzed by ImageJ software.

### 2.11. Statistical Analysis

Results from all experiments are expressed as the mean ± standard error of the mean. The Mann–Whitney *U* test was used to compare the two groups. For single-factor comparison experiments with multiple groups, one-way analysis of variance (ANOVA) followed by Dunnett’s post hoc analysis was conducted for the data. Two-way ANOVA followed by Bonferroni post hoc analysis was performed to analyze the results with two factors. Statistical analyses were performed using GraphPad Prism software (version 8.0) (La Jolla, CA, USA). Statistical significance was set at * *p* < 0.05, ** *p* < 0.01, and *** *p* < 0.001.

## 3. Results

### 3.1. PFKFB3 Expression Increases in Cardiac Tissue after MI

**Figure 1:**
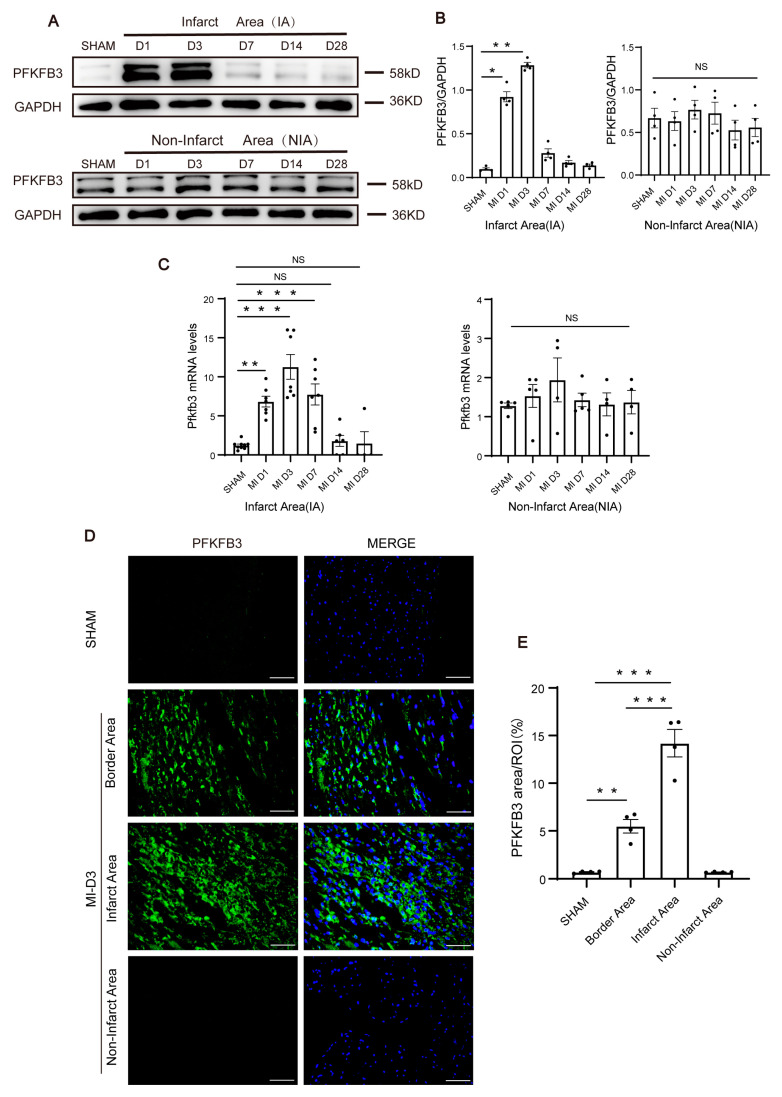
PFKFB3 expression increases in cardiac tissue after myocardial infarction (MI). (**A**,**B**) Protein level detection of Pfkfb3 of murine hearts at days 1, 3, 7, 14, and 28 after MI in both infarct and non-infarct areas (n = 4). (**C**) The mRNA level of Pfkfb3 of murine hearts at days 1, 3, 7, 14 and 28 after MI in both infarct and non-infarct areas (n = 4–9). (**D**,**E**) Representative immunofluorescence staining of Pfkfb3 (green) and DAPI (blue) in murine heart at day 3 after MI, including the infarct area (IA), border area (BA), and non-infarct area (n = 4; scale bar, 50 μm). Data were expressed as mean ± SEM. Data presented in (**B**,**C**,**E**) were analyzed by one-way ANOVA tests. NS indicates not significant. * *p* < 0.05. ** *p* < 0.01. *** *p* < 0.001. DAPI indicates 4′,6-diamidine-2′-phenylindole dihydrochloride; and ROI, region of interest.

To explore whether PFKFB3 is involved in MI, we first measured PFKFB3 expression levels in the myocardium at different time points after MI. PFKFB3 protein and mRNA expression levels increased significantly in the infarct area after MI, with no difference in the non-infarcted area. Specifically, PFKFB3 protein and mRNA expression levels increased on day 1, reached peak values on day 3, and began to decline, approaching baseline levels on day 14 after MI (Figure 1A–C). Immunofluorescence analysis of cardiac tissue from murine LAD models confirmed that PFKFB3 expression increased significantly on day 3 after MI both in the infarct area and the border area, whereas it remained unchanged in the non-infarct area (Figure 1D,E).

### 3.2. 3PO Ameliorates Cardiac Remodeling Induced by MI

**Figure 2:**
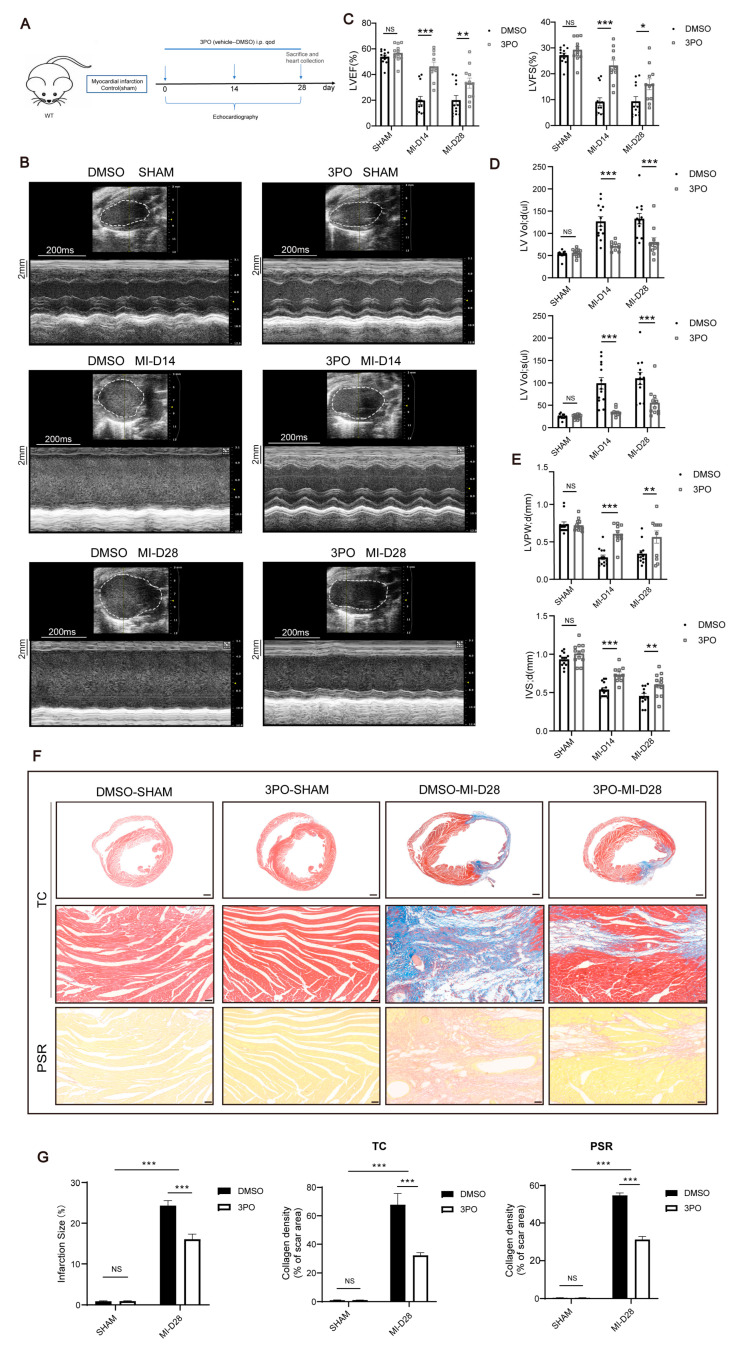
PFKFB3 inhibitor 3PO ameliorates cardiac remodeling induced by myocardial infarction (MI). (**A**) Schematic diagram of the experiment design. (**B**–**E**) Echocardiographic analysis of left ventricular (LV) ejection fraction (EF), LV fractional shortening (FS), LV end-diastolic volume (vol; d), LV end-systolic volume (vol; s), LV posterior wall thickness at diastole (LVPW; d) and interventricular septum thickness at diastole (IVS; d) at days 14 and 28 after MI (n = 9–13) in DMSO and 3PO mice, together with representative B-mode and M-mode echocardiographic images. (**F**) Representative Masson trichrome (TC, top and middle) and picrosirius red (PSR, lower) staining of heart transverse sections obtained from DMSO and 3PO mice at day 28 after MI (scale bar, 500 μm in the top images and 50 μm in the middle and lower images). (**G**) Quantitative analysis of infarction size (left) and collagen density (middle and right) in DMSO and 3PO mice at day 28 after MI (n = 4–6). Data were expressed as mean ± SEM. Data presented in (**C**–**E**,**G**) were analyzed by two-way ANOVA tests. NS indicates not significant. * *p* < 0.05. ** *p* < 0.01. *** *p* < 0.001. DMSO indicates Dimethylsulfoxide; 3PO, 3-(3-pyridinyl)-1-(4-pyridinyl)-2-propen-1-one; and WT, wild type.

To clarify the role of PFKFB3 in the progression of MI, WT mice were i.p. injected with 3PO or DMSO after MI, once every other day. We investigated the cardiac function of mice in the two groups on days 0, 14, and 28 after MI using cardiac ultrasound examination (Figure 2A). We recorded the type B and type M ultrasound images and measured the LV end-diastolic volume [vol(d)], LV end-systolic volume [vol(s)], LV posterior wall thickness at diastole [LVPW(d)], and interventricular septum thickness at diastole [IVS(d)], and we calculated the LV ejection fraction (EF) and LV fractional shortening (FS) at the specified time points. We observed no difference between the two groups at baseline. Both LV vol(d) and LV vol(s) were significantly increased in the MI group compared with the values in the sham-operated groups, whereas EF, FS, LVPW(d), and IVS(d) were considerably decreased. However, 3PO treatment could lead to a significant decrease in LV vol(d) and LV vol(s) and an increase in LVEF and LVFS, accompanied by significant improvements in LVPW(d) and IVS(d), compared with the results of DMSO treatment on days 14 and 28 after MI (Figure 2B–E). In addition, mice in the 3PO group had a smaller LV cavity and a thicker LV wall than those in the DMSO group (Figure 2B,F). The ratio of heart weight to body weight between the DMSO and 3PO groups was examined and we discovered no difference between the two groups (Figure S1A).

Next, we compared the structural changes in the heart between the two treatment groups following MI using Masson trichrome and picrosirius red staining. Masson’s trichrome staining of transverse sections of the heart revealed that the infarction size was significantly smaller in the 3PO group than in the DMSO group. Masson trichrome and picrosirius red staining of the heart infarction area revealed that collagen density in the infarct area was significantly lower in mice treated with 3PO than in those treated with DMSO (Figure 2F,G). Additionally, we applied the TUNEL staining assay and isolectin B4 immunofluorescence staining to examine the impact of 3PO on cardiomyocyte apoptosis and angiogenesis after MI. We discovered that 3PO had no impact on these processes (Figure S1B–E).

### 3.3. 3PO Attenuates Cardiac Fibrosis Induced by MI

**Figure 3:**
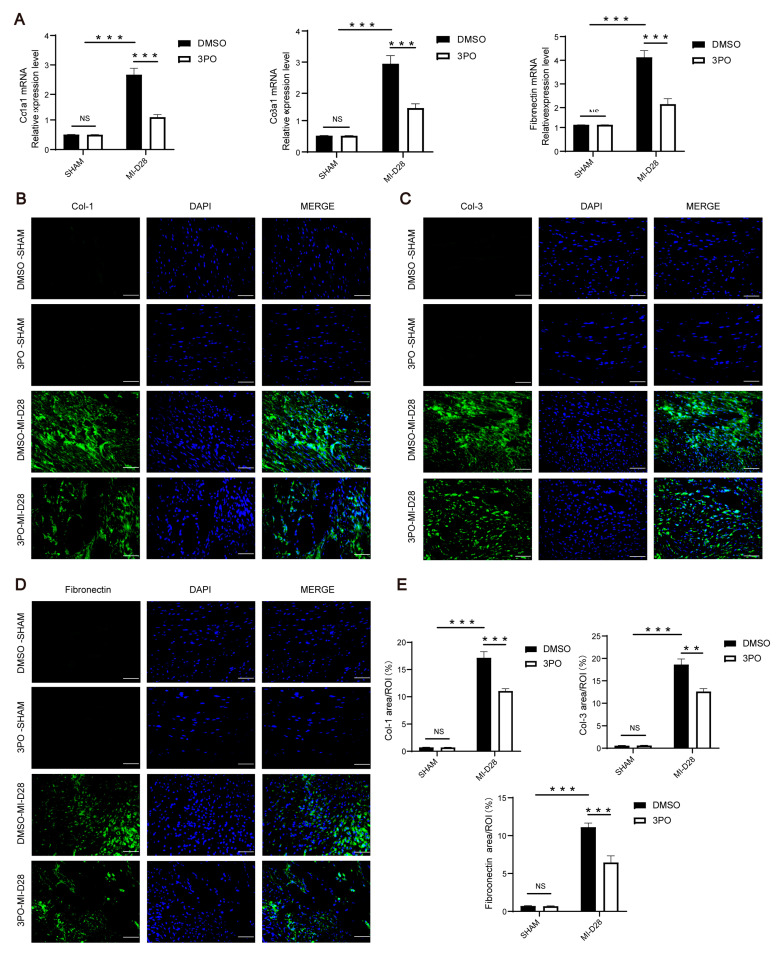
PFKFB3 inhibitor 3PO attenuates cardiac fibrosis induced by myocardial infarction (MI). (**A**) Total mRNA of whole murine hearts was isolated to compare the expression of Col1a1, Col3a1, and fibronectin between DMSO and 3PO mice at day 28 after MI (n = 4–7). (**B**–**E**) Representative immunofluorescence staining of col-1, col-3, fibronectin (all indicated in green), and DAPI (blue) in hearts of DMSO and 3PO mice at day 28 after MI (n = 3–4; scale bar,50 μm). Data were expressed as mean ± SEM. Data presented in (**A**,**E**) were analyzed by two-way ANOVA tests. NS indicates not significant. ** *p* < 0.01. *** *p* < 0.001. DMSO indicates Dimethylsulfoxide; 3PO, 3-(3-pyridinyl)-1-(4-pyridinyl)-2-propen-1-one; Col-1, collagen I; Col-3, collagen III; FN, fibronectin; DAPI, 4′,6-diamidine-2′-phenylindole dihydrochloride; and ROI, region of interest.

The profibrotic reaction gradually begins on day 3 after MI, with collagen deposition following myocyte necroptosis [3]. However, in the infarct area, myofibroblasts secrete a large amount of extracellular matrix, including collagen and fibronectin, which can aggravate poor cardiac remodeling [5]. To explore the effect of 3PO on myocardial fibrosis after MI, we examined the expression of collagen and fibronectin in cardiac tissues 28 days after MI. MI stress led to significant upregulation of collagen I, collagen III, and fibronectin; however, their expression levels were significantly suppressed by 3PO (Figure 3). Additionally, heart sections were stained with antibodies against Gr-1 and α-SMA to investigate the influence of 3PO on the abundance of neutrophils and myofibroblasts after MI. The results indicated that there were no statistical differences in the abundance of neutrophils and myofibroblasts between DMSO and 3PO groups after MI (Figure S2).

### 3.4. 3PO Decreases Expression of PFKFB3 and Inhibits TGF-β1/SMAD2/3 Pathway in Cardiac Tissue after MI

**Figure 4:**
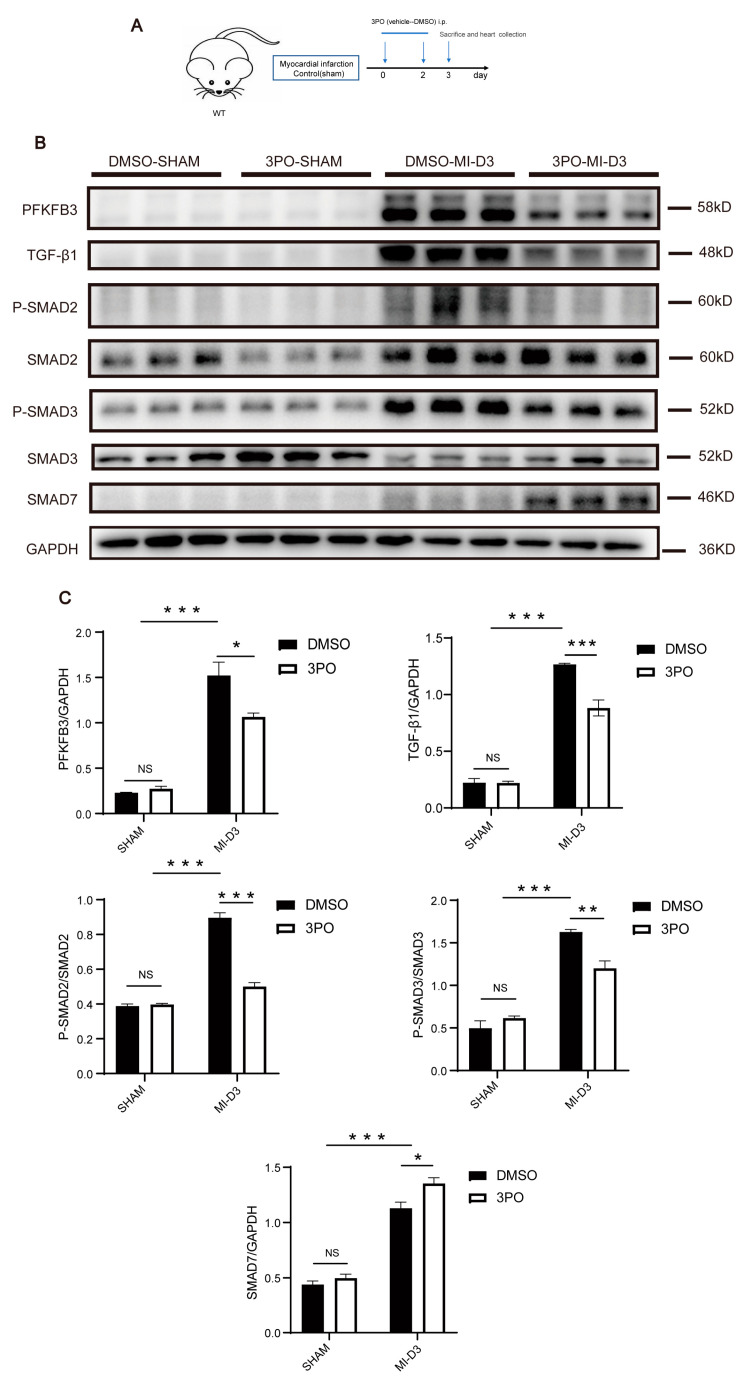
3PO decreases expression of PFKFB3 and inhibits TGF-β1/Smad2/3 pathway after MI. (**A**) Schematic diagram of the experiment design. (**B**,**C**) Western blot analysis of PFKFB3, TGF-β1, P-SMAD2, SMAD2, P-SMAD3, SMAD3, and SMAD7 expression in the DMSO and 3PO mouse hearts at day 3 after MI (n = 3–4). Data were expressed as mean ± SEM. Data presented in (**C**) were analyzed by two-way ANOVA tests. NS indicates not significant. * *p* < 0.05. ** *p* < 0.01. *** *p* < 0.001. DMSO indicates Dimethylsulfoxide; 3PO, 3-(3-pyridinyl)-1-(4-pyridinyl)-2-propen-1-one; and TGF-β1, transforming growth factor-β1.

As a selective inhibitor of PFKFB3, 3PO has been widely used in a variety of studies and has been shown to influence the protein expression of PFKFB3 in vitro [29,38]. To explore the effect of 3PO on MI-induced PFKFB3 protein expression, we treated mice with 3PO or DMSO following MI and detected the expression of PFKFB3 in murine LV tissue on day 3 following MI (Figure 4A). As shown in Figure 4B,C, 3PO significantly reduced the protein expression of PFKFB3 after MI.

TGF-β1/Smad2/3 signaling plays an important role in collagen production and the regulation of cardiac fibrosis following MI. Under the stimulation of MI, TGF-β1 synthesis and secretion increase, as does SMAD2 and SMAD3 phosphorylation [5,15]. In contrast, SMAD7 expression increases to balance the activation of this pathway [39]. To determine whether 3PO might influence this signaling pathway, we detected the expression of these associated proteins in murine LV tissue on day 3 following MI. We found that the expression levels of TGF-β1, P-SMAD2, and P-SMAD3 were significantly decreased, whereas that of SMAD7 was significantly increased in 3PO mice compared with DMSO mice (Figure 4B,C), suggesting that the mechanism by which 3PO protects against myocardial fibrosis is related to inhibition of the TGF-β1/SMAD2/3 signaling pathway.

### 3.5. PFKFB3 Is Abundantly Expressed in CFs following MI and under Hypoxia

**Figure 5:**
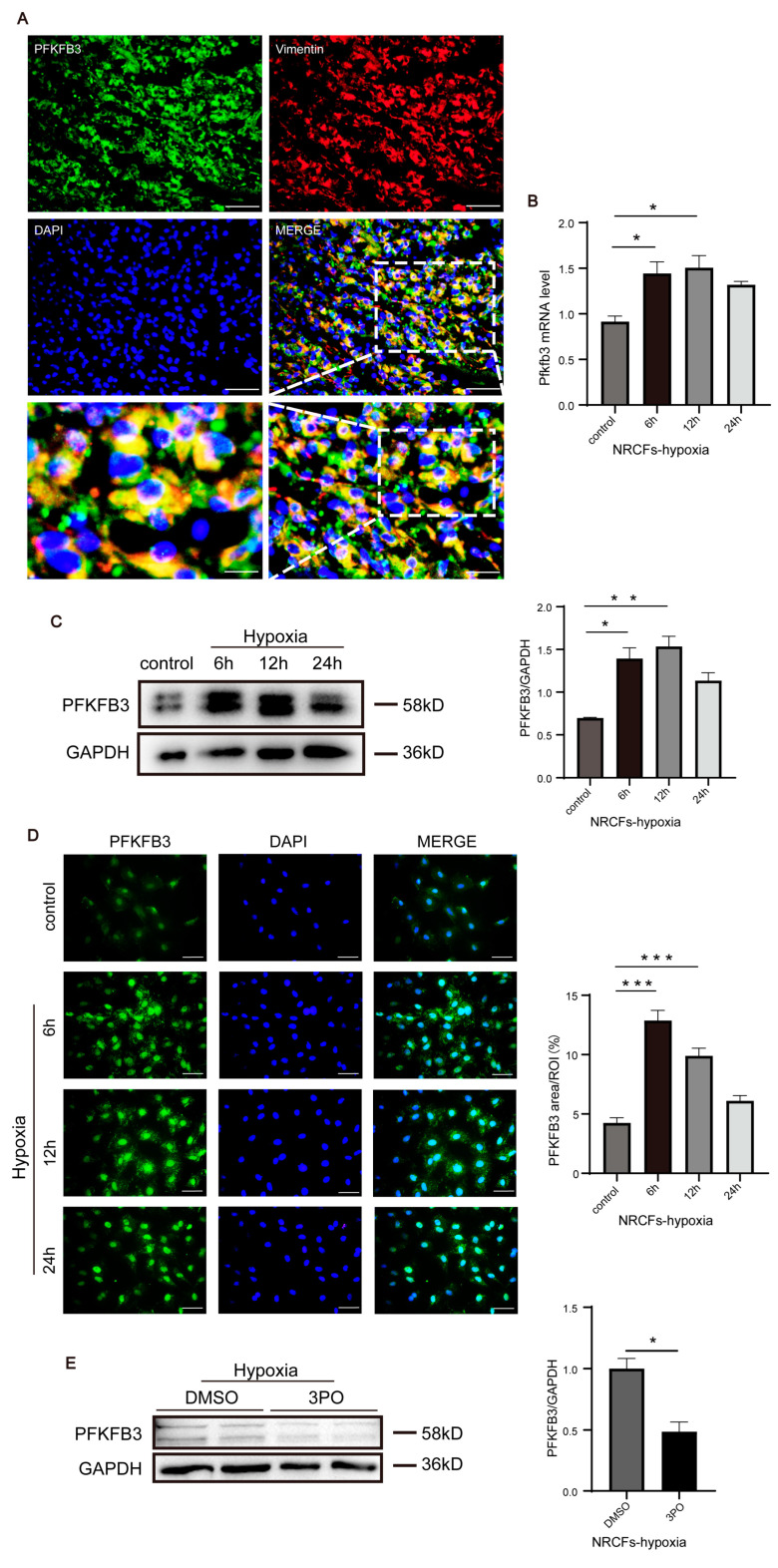
PFKFB3 is abundantly expressed in cardiac fibroblast after MI and under hypoxia. (**A**) Representative dual-immunofluorescence staining of PFKFB3 (green) and Vimentin (red) in murine hearts at day 3 after MI (scale bar, 50 μm in the top and middle images, 12.5 μm in the lower left image, and 25 μm in the lower right image). (**B**,**C**) The mRNA level and protein level of PFKFB3 expression of cardiac fibroblasts treated under hypoxia for 6, 12, and 24 h, respectively (n = 3). (**D**) Representative immunofluorescence staining of Pfkfb3 (green) and DAPI (blue) of cardiac fibroblasts treated under hypoxia for 6, 12, and 24 h, respectively (n = 3–4, scale bar, 50 μm). (**E**) Cardiac fibroblasts were under hypoxia for 6 h after being treated with 3PO (30 μM) or DMSO for 1 h, and the protein levels of PFKFB3 expression were demonstrated by Western blot analysis (n = 4). Data were expressed as mean ± SEM. Data presented in (**B**–**D**) were analyzed by one-way ANOVA tests. Data presented in (**E**) was analyzed by Mann–Whitney *U* tests. NS indicates not significant. * *p* < 0.05. ** *p* < 0.01. *** *p* < 0.001. DMSO indicates Dimethylsulfoxide; 3PO, 3-(3-pyridinyl)-1-(4-pyridinyl)-2-propen-1-one; DAPI, 4′,6-diamidine-2′-phenylindole dihydrochloride; and ROI, region of interest.

Double-immunofluorescence staining of PFKFB3 and vimentin indicated that PFKFB3 was abundantly expressed in CFs following MI (Figure 5A), consistent with its elevated expression in the myocardium on day 3 following MI. Next, we isolated NRCFs and found that PFKFB3 mRNA and protein levels were significantly increased as early as 6 h following the initiation of hypoxia, increased progressively until 12 h, and gradually decreased thereafter (Figure 5B–D). In addition, to explore whether 3PO inhibits PFKFB3 expression in CFs under hypoxia, NRCFs were treated with 30 μM 3PO and then put under hypoxia for 6 h. We found that 3PO decreased the protein level of PFKFB3 in NRCFs under hypoxia (Figure 5E). Together, these results suggested that PFKFB3 may play a role in CFs following MI, further influencing cardiac fibrosis and adverse remodeling.

### 3.6. 3PO Mitigates the Migration, Proliferation, and Activation of CFs Induced by TGF-β1

**Figure 6:**
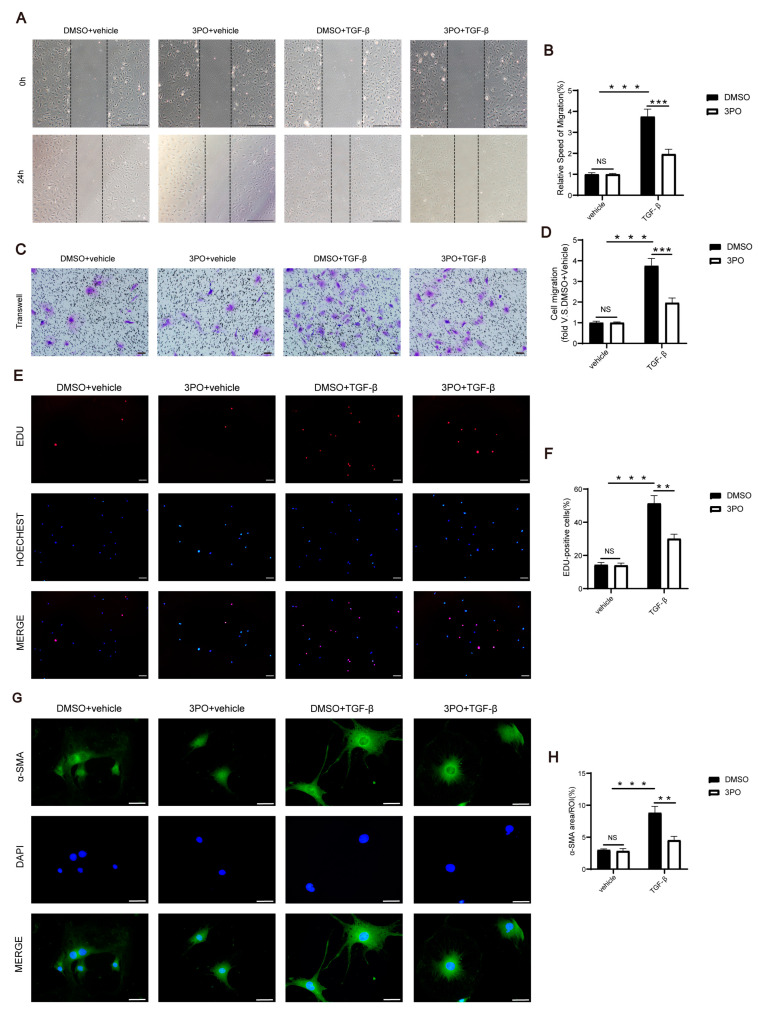
3PO mitigates the migration, proliferation, and activation of CFs induced by TGF-β1. (**A**,**B**) Cardiac fibroblasts were treated with 3PO (30 μM) or DMSO for 1 h and stimulated with TGF-β1 (20 ng/mL) or vehicle for 24 h, and the relative speed of migration was measured by wound healing assay (n = 3–4; scale bar, 1 mm). (**C**,**D**) Cardiac fibroblasts were treated with 3PO (30 μM) or DMSO for 1 h and stimulated with TGF-β1 (20 ng/mL) or vehicle for 24 h, and cell migration was measured by Boyden chamber migration assay (n = 5; scale bar, 100 μm). (**E**,**F**) Cardiac fibroblasts were treated with 3PO (30 μM) or DMSO for 1 h and stimulated with TGF-β1 (20 ng/mL) or vehicle for 24 h, and the proliferation capacity was measured by EDU staining assay (n = 4; scale bar, 100 μm). (**G**,**H**) Cardiac fibroblasts were treated with 3PO (30 μM) or DMSO for 1 h and stimulated with TGF-β1 (20 ng/mL) or vehicle for 24 h, and expression of α-SMA (green) was demonstrated by immunofluorescence staining to indicate fibroblast activation (n = 4; scale bar, 50 μm). Data were expressed as mean ± SEM. Data presented in (**B**,**D**,**F**,**H**) were analyzed by the two-way ANOVA tests. NS indicates not significant. ** *p* < 0.01. *** *p* < 0.001. DMSO indicates Dimethylsulfoxide; 3PO, 3-(3-pyridinyl)-1-(4-pyridinyl)-2-propen-1-one; TGF-β1, transforming growth factor β1; EDU, 5-Ethynyl-2’-deoxyuridine; α-SMA, α-smooth muscle actin; DAPI, 4′,6-diamidine-2′-phenylindole dihydrochloride; and ROI, region of interest.

Upon initiation of the proliferative phase of MI in the heart, fibroblast migration, proliferation, and activation are also triggered [5]. To investigate the effect of 3PO on the migration, proliferation, and activation of CFs induced by TGF-β1, NRCFs were treated with 30 μM 3PO for 30 min and then with 20 ng/mL TGF-β1 for 24 h. Wound healing analysis and the Boyden chamber migration assay were used to explore the migration ability of NRCFs induced by TGF-β1. The results showed that 3PO treatment significantly inhibited TGF-β1-induced NRCF migration (Figure 6A–D). The EDU-positive cells analysis represented that 3PO treatment significantly inhibited TGF-β1-induced NRCF proliferation (Figure 6E,F). Immunofluorescence analysis of α-SMA indicated that 3PO treatment also significantly inhibited TGF-β1-induced NRCF activation (Figure 6G,H).

## 4. Discussion

To our knowledge, this represents the first report of the protective role of 3PO treatment in cardiac remodeling following MI in mice. The expression of PFKFB3 was significantly upregulated in the infarct and border areas in the early phase post-MI. Alternatively, 3PO treatment improved cardiac remodeling and reduced MI-induced excessive cardiac fibrosis by suppressing the TGF-β1/SMAD2/3 signaling pathway. Additionally, PFKFB3 was highly expressed in CFs both following MI and under hypoxic conditions, whereas its inhibition by 3PO attenuated TGF-β1-induced fibroblast migration, proliferation, and activation. Together, these results reveal the potential utility of 3PO treatment for the prevention of long-term heart failure following MI.

PFKFB3, a crucial glycolysis-regulating enzyme, is widely expressed in human tissues and has been shown to affect the pathogenic activity of diverse tissues [40,41]. Recent studies have shown that PFKFB3 expression is markedly enhanced in several animal models of disease, such as acute kidney injury, acute lung injury, and sepsis, and the underlying mechanisms have been investigated [42,43,44]. However, the role of PFKFB3 in ischemic cardiovascular diseases remains uncertain. Here, we describe for the first time that PFKFB3 expression increases significantly in the infarcted and border myocardium following MI, peaks on the third day, and then gradually decreases after seven days, suggesting that PFKFB3 might be essential in the early stages of MI and that its effect might last through the later stages.

LV remodeling following MI usually continues for weeks or months, which results in changes to the structure, size, and function of the LV and plays a crucial role in the development of heart failure. In addition, LV remodeling affects the entire cavity and is linked to the growth of the chambers and time-dependent changes in the form of the heart, from elliptic to spheric [45,46]. To investigate the function of PFKFB3 in long-term ventricular remodeling following MI, we used 3PO, a specific small molecule inhibitor of PFKFB3. On the 14th day after MI, 3PO markedly enhanced systolic function, greatly reduced LV enlargement, and maintained the thickness of the LV wall. Additionally, 3PO preserved the elliptical form of the left heart, whereas the ventricles of the DMSO-treated group had acquired a spherical shape. Similar differences were also observed between the two groups on day 28. Furthermore, we demonstrated that 3PO reduced cardiac fibrosis and collagen deposition in infarcted and border zones. According to a previous study that supported our findings, 3PO treatment reduced tumor necrosis factor-induced nuclear factor-κB translocation to the nucleus in liver sinusoidal endothelial cells and attenuated liver fibrosis in a murine early liver fibrosis model [47]. Henderson et al. [48] found that 3PO attenuated the TGF-β1-driven increase in collagen I of the dermal fibroblasts. Moreover, Xu et al. [49] discovered that 3PO downregulated the protein expression of collagen I and α-SMA in human fetal lung fibroblasts. These research results indicate that 3PO has a lot of potentials to be studied in the fibrosis process, and it is important to look into the mechanism underlying 3PO’s function in this process. PFKFB3 expression levels increased on day 3 after MI and then declined in the late post-infarction period, and continuous inhibition of its function by 3PO significantly led to improved cardiac function and fibrotic remodeling on day 28 after MI. A prior study [50] found that Dectin-2 expression increased on day 3 after MI and declined in a few days, and Dectin-2 knockout led to an improvement in cardiac function, which is similar to our findings. Another prior research study found that IL-13 expression peaked on day 3 after MI and IL-13 deficiency aggravated cardiac remodeling [51]. These phenomena implied that the early post-MI elevated molecule also has considerable effects on myocardial remodeling and cardiac function in later stages. However, 3PO may influence ventricular remodeling in late infarction independently of its target PFKFB3, which should be more fully explored in future studies. Overall, our results confirm that 3PO treatment could reduce excessive fibrosis and improve cardiac function following MI.

A study demonstrated that TGF-β1 mRNA levels increased after 12 h following MI, reached a maximum on day 3, and then decreased and remained significantly elevated at two- to three-fold [10]. When TGF-β1 protein is synthesized at high levels, a cascade response occurs that extremely activates downstream SMAD2/3 signaling pathways. TGF-β1 signaling through SMAD2/3 pathways [52] is essential for the induction and maintenance of the activated fibroblast phenotype and fibroblast proliferation associated with the post-MI phase [5,6]. Feng et al. [14] and Li et al. [15] discovered that inhibition of the TGF-β1/SMADs signaling pathway significantly reduced cardiac fibrosis following MI. Hu et al. [29] demonstrated that the administration of 3PO reduced the expression of PFKFB3 in lung tissues, which is upregulated in reaction to LPS stimulation. In the present study, we found that 3PO decreased the expression of PFKFB3 and TGF-β1 in the myocardium and hindered the production of collagen and fibronectin by suppressing the TGF-β1/SMAD2/3 pathway following MI. Platelets, leukocytes, and fibroblasts in the infarcted myocardium can synthesize and secrete TGF-β1, and the production of reactive oxygen species (ROS), activation of proteases, and induction of extracellular matrix proteins will release and activate it in large quantities [5,53]. The decline in TGF-β1 protein expression by 3PO might be thus mediated by inhibition of ROS production, extracellular matrix induction, and protease activation, or by directly reducing TGF-β1 synthesis in cells. Reduced TGF-β1 binding to TGFBR or other yet-to-be-studied pathways might also be the cause of 3PO-mediated inhibition of the downstream SMAD2/3 pathway. Xie et al. [33] reported that PFKFB3 inhibition prevents the activation of pulmonary fibroblasts; therefore, we hypothesized that PFKFB3 also plays a role in CFs. Consistent with this, we discovered that PFKFB3 was abundantly expressed in fibroblasts in the infarction zone on the third day following MI, and that cellular hypoxia stimulated the expression of PFKFB3 in NRCFs, peaking at 12 h. Recently, Schoors et al. [54] reported that 3PO reduced the proliferation and migration of endothelial cells. A different study showed that 3PO dramatically reduced the migratory and proliferative activity of ovarian cancer cells [55]. Here, we demonstrated that 3PO reduced the migration, proliferation, and activation capacity of NRCFs following TGF-β1 stimulation. However, additional studies are needed to elucidate the specific mechanism through which 3PO suppresses the migration, proliferation, and activation capacity of NRCFs.

As a regulator of glycolytic rate-limiting enzymes, PFKFB3 is a popular molecule in tumor-related research [56,57]. In the present study, we showed that PFKFB3 is involved in MI, and that inhibition of PFKFB3 using 3PO ameliorated ventricular remodeling and cardiac function. Nevertheless, our study had some limitations. First, we did not use PFKFB3 knockout or silencing in combination with overexpression strategies in the in vivo and in vitro models to establish the bidirectional validation of the role of this protein in MI. Second, we did not examine whether the protective impact of 3PO on cardiac remodeling following MI depends on dose and time. Third, further investigation is required to determine whether 3PO can exert its protective effects via mechanisms other than PFKFB3 inhibition. Fourth, we did not investigate whether 3PO has an impact on other body tissues and how best to target 3PO to the heart. Fifth, more experiments should be conducted to clarify the potential mechanism by which PFKFB3 regulates the TGF-β/SMADs signaling pathway. These limitations provide important directions for our future research.

## 5. Conclusions

In conclusion, we demonstrated that 3PO played a protective role following MI by reducing cardiac fibrosis and alleviating ventricular remodeling. Our study suggests that 3PO treatment may be a potential therapeutic target to prevent cardiac remodeling following MI and lower the incidence of chronic heart failure.

## Data Availability

The raw data supporting the conclusions of this article will be made available by the authors, without undue reservation.

## Supplementary Materials

The following supporting information can be downloaded at https://www.mdpi.com/article/10.3390/biom13071072/s1, Table S1: List of the antibodies and other reagents used in this study; Table S2: Echocardiographic analysis of DMSO and 3PO mice at days 14 and 28 after MI or sham surgery; Table S3: Primers for real-time PCR analysis in mice and cells; Figure S1: 3PO has no impact on the ratio of heart weight to body weight, cardiomyocyte apoptosis, or angiogenesis after MI; Figure S2: 3PO has no influence on the abundance of neutrophils and myofibroblasts after MI; Figure S3: Original membranes of Western blotting analysis of PFKFB3 after MI; Figure S4: Original membranes of Western blotting analysis of PFKFB3, TGF-β1, SMAD2 phosphorylation, SMAD3 phosphorylation, and SMAD7 at day 3 after MI; Figure S5: Original membranes of Western blotting analysis of PFKFB3 of Neonatal Rat Cardiac Fibroblasts (NRCFs) under hypoxia.
