# Supplementary material for: PFKFB3 Inhibitor 3PO Reduces Cardiac Remodeling after Myocardial Infarction by Regulating the TGF-β1/SMAD2/3 Pathway
Source: Biomolecules. 2023 Jul 3;13(7):1072. doi: 10.3390/biom13071072 (PMC10377206; doi:10.3390/biom13071072)

Supplemental Material:

PFKFB3 inhibitor 3PO reduces cardiac remodeling after myocardial infarction by regulating the TGF-β1/SMAD2/3 pathway.

Qian Yang 1,2,†, Xiao Zong 1,2,†, Lingfang Zhuang 1,2, Roubai Pan 1, Xierenayi Tudi 1,2, Qin Fan 1,*, and Rong Tao 1,*

Table S1. List of the antibodies and other reagents used in this study.

Antibody specificity	Company	Cat. No.	Application	Dilution
**Mouse antibody for Western Blot**				
PFKFB3	abcam	ab181861	WB	1:1000
GAPDH	Proteintech	60004-1-1g	WB	1:3000
P-SMAD2	Cell Signaling Technology	18338	WB	1:1000
SMAD2	Cell Signaling Technology	5339	WB	1:1000
P-SMAD3	abcam	ab52903	WB	1:1000
SMAD3	abcam	ab40854	WB	1:1000
SMAD7	Santa Cruz Biotechnology	Sc-365846	WB	1:1000
TGF-β	abcam	ab179695	WB	1:1000
**Secondary antibody for Western Blot**				
Anti-rabbit	Cell Signaling Technology	7074	WB	1:3000
Anti-mouse	Cell Signaling Technology	7076	WB	1:3000
**Mouse antibody for IF**				
PFKFB3	Proteintech	13763-1-AP	IF	1:100
COL-1	abcam	ab34710	IF	1:100
COL-3	abcam	ab7778	IF	1:100
FN	abcam	ab2413	IF	1:100
VIMENTIN	Servicebio	GB12192	IF	1:100
DAPI	Beyotime Biotechnology	C1006	IF	1:1000
α-SMA	abcam	ab28052	IF	1:100
α-actinin	Servicebio	GB11555	IF	1:500
Gr-1	Servicebio	GB11229	IF	1:200
Isolectin B4	Vector	B-1205	IF	1:100

**Rat antibody for IF**				
PFKFB3	Proteintech	13763-1-AP	IF	1:100
α-SMA	abcam	ab28052	IF	1:100
DAPI	Beyotime Biotechnology	C1006	IF	1:1000
**Secondary antibody conjugated with fluorescence**				
Goat anti-Rabbit Secondary Antibody-Alexa Fluor® 488	Thermo Fisher	A11034	IF	1:1000
Goat anti-Rabbit Secondary Antibody-Alexa Fluor® 555	Thermo Fisher	A21428	IF	1:1000
Goat anti-Mouse Secondary Antibody-Alexa Fluor® 488	Thermo Fisher	A11001	IF	1:1000
Goat anti-Mouse Secondary Antibody-Alexa Fluor® 488	Thermo Fisher	A21422	IF	1:1000

**PFKFB3 inhibitor and vector used in mouse**				
3PO	MCE	HY-19824	inhibitor	3.5mg/ml
DMSO	MCE	HY-Y0320	vector	5:7

**PFKFB3 inhibitor, Rat Recombinant protein, and their vectors used in cell**				
3PO	MCE	HY-19824	inhibitor	30μM
DMSO	MCE	HY-Y0320	vector	1:1000
Recombinant Human TGF-β Protein	RD	240-B-002	Recombinant protein	20ng/ml
Reconstitution Buffer 4 (BSA/HCl)	RD	RB04	vector	1:1

Table S2. Echocardiographic analysis of DMSO and 3PO mice at days 14 and 28 after MI or sham surgery.

Group	SHAM		MI-D14		MI-D28	
	DMSO	3PO	DMSO	3PO	DMSO	3PO
n	13	12	13	10	12	11
HR(beats/min)	471.26±4.43	467.26±4.00	472.3±4.49	491.88±5.13	481.00±4.79	485.35±4.65
EF(%)	53.80±1.38	56.78±1.88	19.83±3.14	46.31±3.42***	20.01±3.71	33.39±3.94$^{\#\#}$
FS(%)	27.19±0.83	29.27±1.23	9.17±1.54	23.21±2.09***	9.36±1.83	15.97±2.12$^{\#}$
Corrected LV mass(mg)	82.3±3.00	90.46±2.93	67.31±7.23	70.14±6.54	62.23±4.09	66.67±5.51
LV Vol ; d(μl)	53.31±2.12	56.67±2.39	126.75±10.65	71.37±3.22***	133.10±11.88	80.49±9.81$^{\#\#\#}$
LV Vol ; s(μl)	24.8±1.45	24.45±1.37	99.08±12.63	34.5±2.69***	110.01±13.47	55.49±9.56$^{\#\#\#}$
LVPW; d(mm)	0.73±0.03	0.72±0.02	0.29±0.03	0.61±0.04***	0.34±0.04	0.57±0.08$^{\#\#}$
IVS; d(mm)	0.93±0.02	1.01±0.04	0.54±0.03	0.74±0.03***	0.46±0.03	0.61±0.05$^{\#\#}$

Data are expressed as mean ± SEM and analyzed using the two-way ANOVA test. *Statistical difference between DMSO and

3PO at day 14 after MI. $^{*}P<0.05$. $^{**}P<0.01$. $^{***}P<0.001$. $^{\#}$Statistical difference between DMSO and 3PO at day 14 after MI. $^{\#}P<0.05$. $^{\#\#}P<0.01$. $^{\#\#\#}P<0.001$.

DMSO= Dimethylsulfoxide; 3PO= 3-(3-pyridinyl)-1-(4-pyridinyl)-2-propen-1-one; MI= myocardial infarction; HR=heart rate; EF= ejection fraction; FS= fractional shortening; LV Vol ; d= left ventricular end-diastolic volume; LV Vol ; s= left ventricular end-systolic volume; LVPW; d= left ventricular posterior wall thickness at diastole; IVS; d= interventricular septum thickness at diastole.

Table S3. Primers for real-time PCR analysis in mice and cells.

GENE	Forward	Reverse
**Primers for mice**		
Pfkfb3	CAACTCCCCAACCGTGATTGT	GAGGTAGCGAGTCAGCTTCTT
Gapdh	AGGTCGGTGTGAACGGATTTG	TGTAGACCATGTAGTTGAGGTCA
Col1a1	GCTCCTCTTAGGGGCCACT	CCACGTCTCACCATTGGGG
Col3a1	ACGTAGATGAATTGGGATGCAG	GGGTTGGGGCAGTCTAGTG
Fibronectin	GCTCAGCAAATCGTGCAGC	CTAGGTAGGTCCGTTCCCACT
**Primers for NRCFs**		
Pfkfb3	CGGACAACCTTTGCTAGGGA	TTCTGGGAAGATTCGGCACC
Gapdh	ATGGGAAGCTGGTCATCAAC	GTGGTTCACACCCATCACAA

Figure S1. 3PO has no impact on the ratio of heart weight to body weight, cardiomyocyte apoptosis, or angiogenesis after MI. (A)Summarized data of heart weight/body weight (HW/BW) for sham- and MI-operated mice at day 28 after surgery (n=3-5). (B)and(D)The TUNEL assay (red), α-actinin (green), and DAPI (blue) immunofluorescence co-staining were used to determine cardiomyocyte apoptosis in infarct areas and border areas between DMSO and 3PO mice at day 1 after MI (n=3; scale bar,20 μm). (C) and (E)Representative immunofluorescence staining of IB4 (green) and DAPI (blue) in hearts of DMSO and 3PO mice at day 28 after MI (n=4; scale bar,50 μm). Data were expressed as mean ± SEM. Data presented in (D) were analyzed by one-way ANOVA tests. Data presented in (E) were analyzed by the Mann-Whitney *U* test. NS indicates not significant. DMSO indicates Dimethylsulfoxide; 3PO, 3- (3-pyridinyl)-1-(4-pyridinyl)-2-propen-1-one; TUNEL, TdT-mediated dUTP Nick-End Labeling; IB4, isolectin B4; DAPI, 4′,6-diamidine-2′-phenylindole dihydrochloride; and ROI, region of interest.

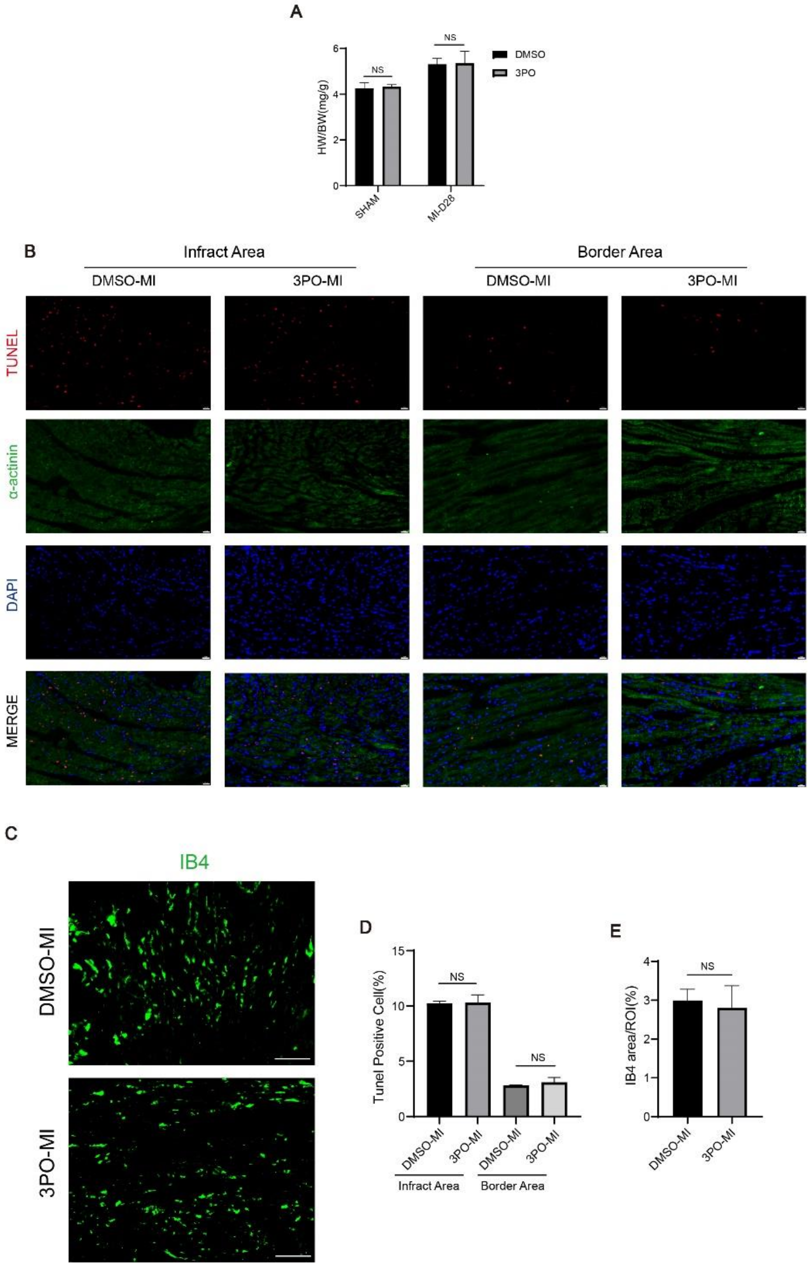

Figure S2. 3PO has no influence on the abundance of neutrophils and myofibroblasts after MI. (A) and (C) Representative immunofluorescence staining of Gr-1 (green) and DAPI (blue) in hearts of DMSO and 3PO mice at day 1 after MI (n=5; scale bar,20 μm). (B) and (D) Representative immunofluorescence staining of α-SMA(green) and DAPI(blue) in hearts of DMSO and 3PO mice at day 28 after MI (n=6; scale bar,50 μm). Data were expressed as mean ± SEM. Data presented in (C) and (D) were analyzed by the Mann-Whitney *U* test. NS indicates not significant. DMSO indicates Dimethylsulfoxide; 3PO, 3- (3-pyridinyl)-1- (4-pyridinyl)-2-propen-1-one; α-SMA, α-smooth muscle actin; and DAPI, 4′,6-diamidine-2′-phenylindole dihydrochloride.

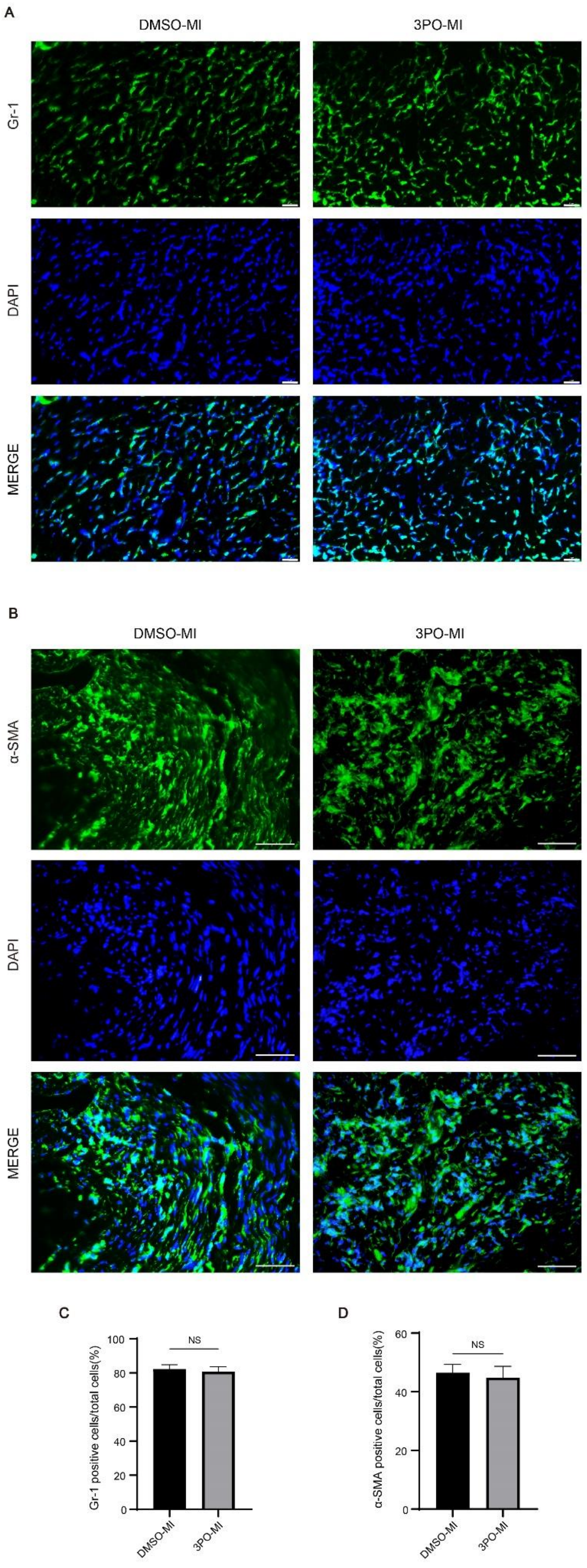

A
DMSO-MI
3PO-MI
Gr-1
DAPI
MERGE
B
DMSO-MI
3PO-MI
α-SMA
DAPI
MERGE
C
Gr-1 positive cells/total cells(%)
NS
0
20
40
60
80
100
DMSO-MI
3PO-MI
D
α-SMA positive cells/total cells(%)
NS
0
20
40
60
DMSO-MI
3PO-MI

Figure S3. Original membranes of western blotting analysis of PFKFB3 after MI. Western Blotting membranes for PFKFB3 and GAPDH of murine hearts at day 1,3,7,14, and 28 after MI in both infarct and non-infarct areas. Total protein extracted from WT mice, n≥3 per group.

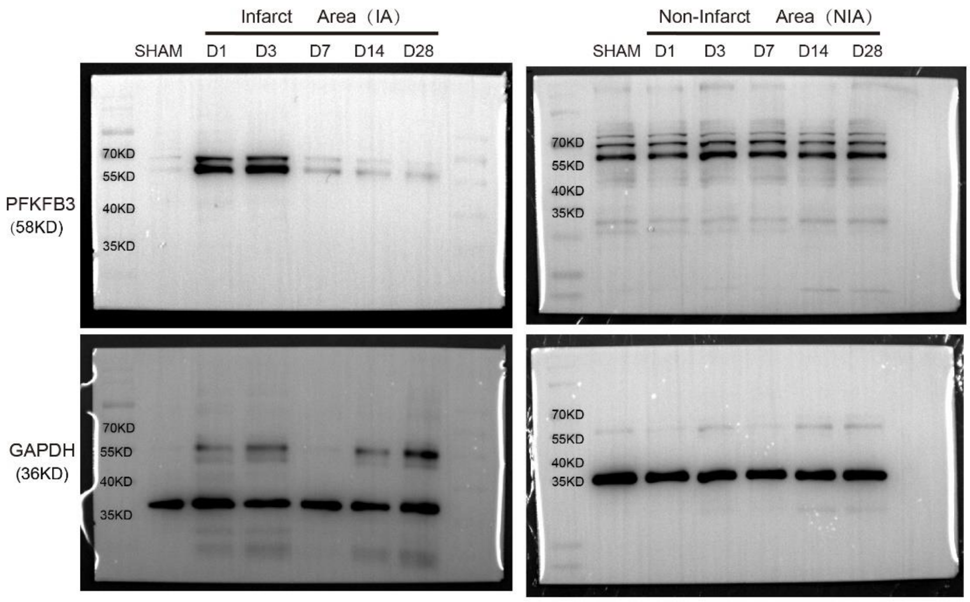

Figure S4. Original membranes of western blotting analysis of PFKFB3, TGF-β1, SMAD2 phosphorylation, SMAD3 phosphorylation, and SMAD7 at day 3 after MI. Western Blotting membranes for PFKFB3, TGF-β1, P-SMAD2, SAMD2, P-SMAD3, SMAD3, SMAD7, and GAPDH of murine hearts at day 3 after MI. Total protein extracted from DMSO and 3PO mice, n≥3 per group.

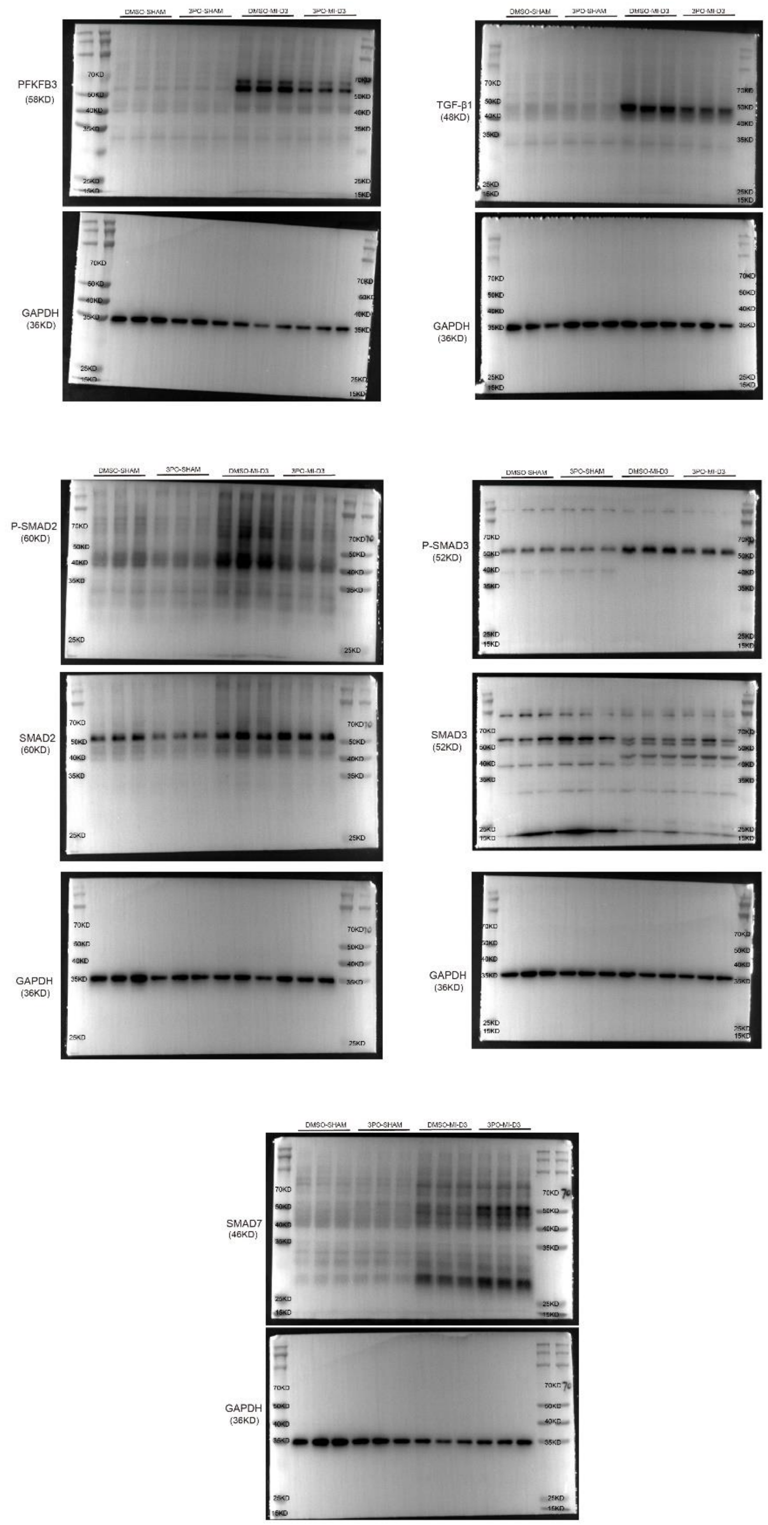

Figure S5. Original membranes of western blotting analysis of PFKFB3 of Neonatal Rat Cardiac Fibroblasts(NRCFs) under hypoxia. Western Blotting membranes on the left for PFKFB3 and GAPDH of NRCFs under hypoxia for 6, 12, and 24h, respectively. Western Blotting membranes on the right for PFKFB3 and GAPDH of NRCFs stimulated by DMSO or 3PO under hypoxia for 6h. Total protein

extracted from NRCFs, n≥3 per group.

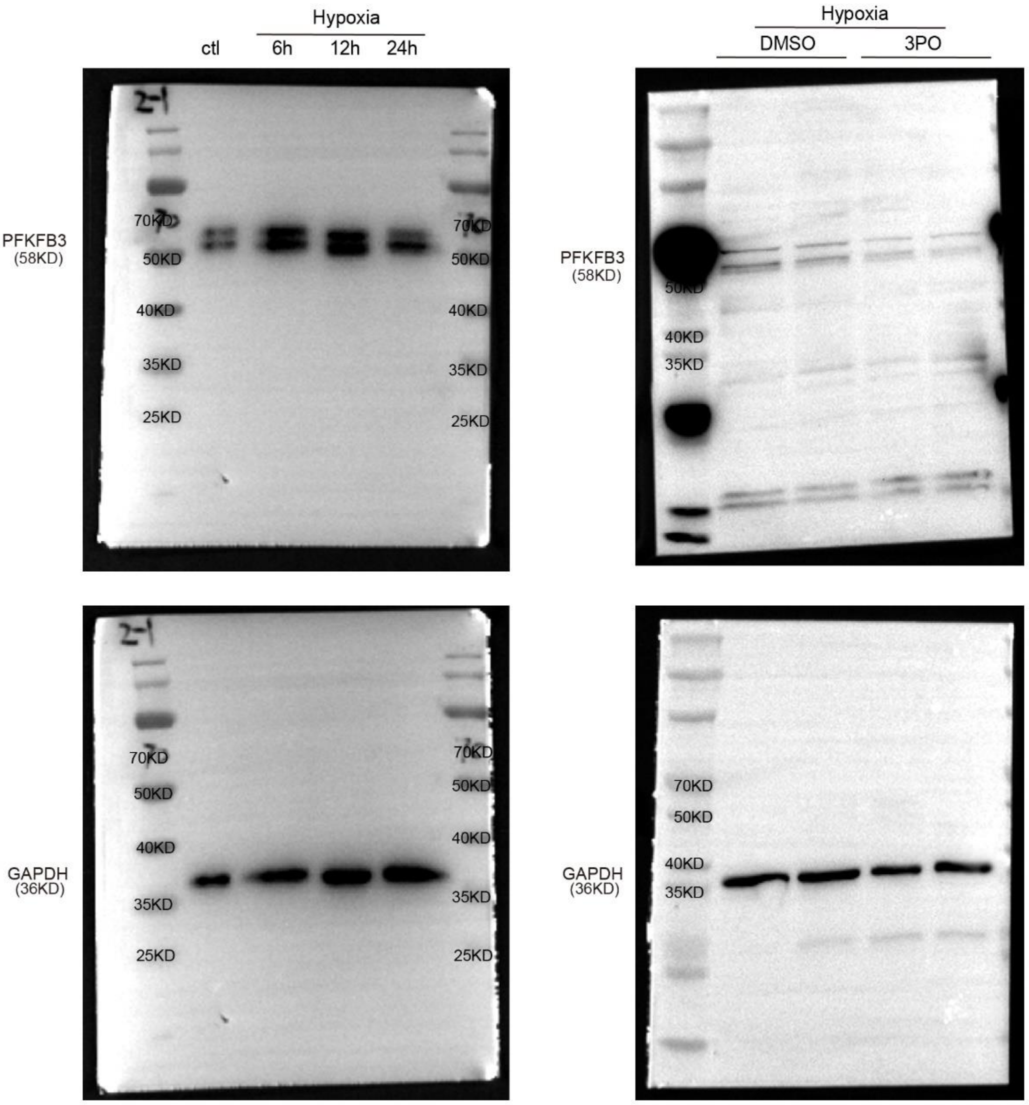

Supplement: Supplementary file 1 [file biomolecules-13-01072-s001.zip › biomolecules-2359801-supplementary.pdf]
